# In Vitro Inactivation of Respiratory Viruses and Rotavirus by the Oral Probiotic Strain *Weissella cibaria* CMS1

**DOI:** 10.1007/s12602-022-09947-z

**Published:** 2022-05-10

**Authors:** Mi-Sun Kang, Geun-Yeong Park

**Affiliations:** R&D Center, OraPharm Inc, Seoul, 04782 Republic of Korea

**Keywords:** Probiotics, Human respiratory syncytial virus, Influenza virus A, Rotavirus A, Inactivation

## Abstract

*Weissella cibaria* CMS1 (oraCMS1) has been commercially used in Korea as an oral care probiotic for several years. Human respiratory syncytial virus (RSV) and the influenza A virus (H1N1) are representative viruses that cause infantile lower respiratory tract infections. Rotavirus A (RVA) is the most common cause of diarrhea in infants and young children. Here, we aimed to evaluate the efficacy of the cell-free supernatant (CFS) of oraCMS1 in inactivating RSV, H1N1, and RVA in suspension as per ASTM (American Society for Testing and Materials) E1052-20. The mixture of oraCMS1 and these viruses was evaluated at contact times of 1, 2, and 4 h. Virucidal activity was measured using a 50% tissue culture infective dose assay (log_10_TCID_50_) after infecting the host cells with the viruses. The CFS of oraCMS1 inactivated RSV by up to 99.0% after 1 h and 99.9% after 2 and 4 h, and H1N1 and RVA were inactivated by up to 99.9% and 99.0% at 2 h, respectively. Although these in vitro results cannot be directly interpreted as implying clinical efficacy, our findings suggest that oraCMS1 provides a protective barrier against RSV, H1N1, and RVA, and therefore, it can help decrease the risk of respiratory tract and intestinal infections.

## Introduction

Acute respiratory viral infections and gastroenteritis are important causes of morbidity and mortality worldwide [1]. Lower respiratory tract infections are responsible for 808,920 deaths in 2017 [2] and rotavirus infection caused 128,500 deaths and 258 million episodes of diarrhea among children younger than 5 years in 2016 [3]. Most respiratory virus (RV) infections are characterized by annual outbreaks in winter and spring [4]. Of approximately 200 RVs, human respiratory syncytial virus (RSV) and the influenza A virus (H1N1) are among the most important viruses associated with hospitalizations and deaths [4–6].

RSV causes common colds and lower respiratory tract infections, such as bronchitis, bronchiolitis, and pneumonia, in children and adults and is the most common cause of pneumonia among children younger than 5 years [7]. RSV is a negative-sense, single-stranded RNA virus, whose name is derived from the large syncytium that forms when infected cells fuse together [8]. Acute otitis media and sinusitis are also caused by RVs [9]. In addition, many studies have shown that RSV is the virus most commonly associated with acute otitis media in infants [9]. Sinusitis occurs in 10% of adult patients with acute RV infections, for which RSV and H1N1 are the most commonly associated viruses [10].

H1N1 is the most common cause of influenza affecting humans, triggering both the Spanish flu in 1919 and the swine flu pandemic in 2009, and has been associated with increased rates of hospitalization and death, particularly among adults [11]. H1N1 is an enveloped single negative-stranded RNA virus that apparently causes more than 500,000 deaths annually worldwide [12].

Rotavirus is the most frequent cause of diarrhea among infants and children. By the age of 5 years, almost all children are infected with rotavirus at least once. While most commonly associated with children, it can also infect adults [13, 14]. Rotaviruses are divided into eight types, designated as groups A to H. Humans are primarily infected by rotavirus groups A, B, and C, whereas pigs are infected by groups E and H, and birds by groups D, E, and F. Among these, rotavirus A (RVA), a naked virus containing double stranded DNA, is the best known [13–15].

Probiotics are live bacteria that enter the body in adequate amounts and exert beneficial effects [16]. For the past 20 years, *Lactobacillus* and *Bifidobacterium* species have played substantial roles as representative probiotics used in dietary supplements. Recently, a specific strain of *Weissella cibaria* has also been identified as an oral probiotic [17]. *W. cibaria* CMS1 is a strain isolated from the saliva of Korean children aged 4–7 years with a healthy oral cavity; this strain was proved safe and could inhibit the formation of the *Streptococcus mutans* biofilm [17–19]. In addition, the antimicrobial and antibiofilm activities of *W. cibaria* CMS1 against major pathogens of upper respiratory tract infections including *Streptococcus pyogenes*, *Streptococcus pneumoniae*, and *Moraxella catarrhalis* have been elucidated recently [20].

*S. mutans* is a representative bacterium that causes caries and plays an important role in the early stages of caries development [21]. *S. mutans* is first detected in the oral cavities of infants from the age of 6 months, usually during the early teething period. This bacterium forms microbiota on the tooth surface, and the number of which gradually increases, with the fastest rate of bacterial growth occurring between 19 and 33 months of age. This period is known as the “window of infectivity” [22]. Together, these studies suggest that consuming *W. cibaria* CMS1 during this period can help prevent dental caries as well as upper respiratory tract infections and enteritis caused by viruses.

To date, antiviral probiotics have not been used in the fields of medicine and food science. Although studies have assessed the effectiveness of various probiotic types in preventing and treating viral infections [23–25], only a few have evaluated the antiviral effects of the oral probiotic *W. cibaria* against RVs and enteric viruses. Here, we present a study demonstrating the in vitro efficacy of the oral probiotic strain *W. cibaria* CMS1 against RSV, H1N1, and RVA.

## Materials and Methods

### Cell-Free Supernatant of oraCMS1

We evaluated the antiviral effects of *W. cibaria* CMS1 (oraCMS1; OraPharm Inc., Seoul, Republic of Korea), which has probiotic properties and is used in oral care [16–18]. *W. cibaria* was grown aerobically in 300 mL de Man, Rogosa, and Sharpe broth (MRS broth; Difco Laboratories, Detroit, MI, USA) at 37 °C for 24 h. The bacterial cell-free supernatant (CFS) was prepared by removing the cells (log 8.43 ± 0.05 colony forming unit (CFU)/mL) by centrifugation (4000 × *g*, 20 min, 4 °C) and filter-sterilizing the supernatant (0.45 µm pore size; Millipore, Burlington, MA, USA). The CFS of oraCMS1 was used as the test solution.

### Cell Lines

Hep-2 (from human laryngeal carcinoma) and CV-1 (from the kidney of a male adult African green monkey) cells were obtained from Korean Cell Line Bank (KCLB; Seoul, Republic of Korea). MDCK (from Madin-Darby canine kidney) cell was obtained from American Type Culture Collection (ATCC; Manassas, VA, USA). Hep-2 (KCLB 10,023), MDCK (ATCC CCL-34), and CV-1 (KCLB 10,070) cells were used as host cells for RSV, H1N1, and RVA, respectively. The cells were maintained at 37 °C in 5% CO_2_ in Eagle’s minimum essential medium (EMEM; ATCC, Manassas, VA, USA) supplemented with 10% fetal bovine serum (Gibco, Thermo Fisher Scientific, Waltham, MA, USA) and 100 U/mL penicillin–streptomycin (Gibco).

### Viral Preparation

The following challenge viruses were investigated: human RSV strain long (ATCC VR-26), H1N1 strain A/PR/8/34 (tissue culture [TC] adapted) (ATCC VR-1469), and RVA strain Wa (TC adapted) (ATCC VR-2018). RSV, H1N1, and RVA were prepared by infecting each host cell line, seeded in T-150 flasks, for 60–90 min. At approximately 3–12 days post-infection, marked cytopathic effects (CPEs) were evident in more than 90% of each host cell type. The culture supernatant was centrifuged at 2000 × *g* for 10 min and filtered (0.45 µm pore size) to isolate the virus. Viral titers of the culture supernatant were measured based on a log 50% TC infective dose (log_10_TCID_50_), and the supernatant was used in the infection experiments.

### Cytotoxicity Test

The viral culture medium (VCM, EMEM containing 2% fetal bovine serum for RSV; EMEM containing 0.3% bovine serum albumin and 1 μg/mL L-(tosylamido-2-phenyl) ethyl chloromethyl ketone-treated trypsin for H1N1; EMEM containing 2 μg/mL trypsin for RVA) was mixed with the test solution at a ratio of 1:9 and serially diluted tenfold with VCM. The diluted test solution was treated with host cells at 37 °C for 30–60 min, and cytotoxicity was visually observed using a microscope. As we observed that the level of cytotoxicity did not decrease upon serial dilution, it was applied to the following tests after filtering with Sephadex LH-20 (Sigma-Aldrich, St. Louis, MO, USA) (ASTM E1482-12) and SCDLP (Difco) as a neutralizing agent.

### Sub-cytotoxicity Test

Each concentration of the test solution that did not show cytotoxicity was treated with host cells for 30 min, and the cell monolayer was washed with phosphate-buffered saline. The diluted test viral solution was infected for 60–90 min and incubated for 3–12 days for RSV, 3–5 days for H1N1, and 1–7 days for RVA.

### Neutralization Test

The neutralized test solution and diluted test viral solution were mixed in equal amounts, allowed to react for 10–20 min, and incubated according to the conditions of viral culture.

### Viral Inactivation Test

The efficacy of viral inactivation for oraCMS1 was tested in accordance with the ASTM E1052-20 method, titled “Standard Practice to Assess the Activity to Microbicides against Viruses in Suspension” [26] at the BSL-2 laboratory of the Korea Testing and Research Institute, Gwacheon-si, Gyeonggi-do, Republic of Korea. Host cells treated with VCM were used as negative control. Briefly, the test virus was mixed with VCM at a ratio of 1:9, serially diluted 1:10, and inoculated into host cells to measure the initial titer of the test virus. After incubation at 37 °C for 1, 2, and 4 h, the solution was filtered using a neutralization method, and the viral titers of the positive viral controls were measured via serial dilutions with VCM. The test virus was mixed with the test solution at a ratio of 1:10 and incubated at 37 °C for 1, 2, and 4 h. Immediately after completion of the reaction, neutralization was performed using the abovementioned neutralization method, followed by serial dilution. Each dilution was applied to the host cells to measure the viral titer in the test group. After the reaction was completed, each solution was serially diluted and added to 96-well plates containing test viral monolayers (*n* = 8 replicates per dilution) and cultured according to each viral culture condition. The cells were treated with 25% methyl alcohol (v/v) and 0.5% crystal violet (w/v) and stained for 10 min at 22 ± 2 °C. The number of stained wells was counted, and TCID_50_ titers for positive viral controls and neutralized oraCMS1 test conditions were determined using the Spearman–Karber method [27]. The log_10_ reduction values achieved by the various oraCMS1 lots, and exposure time points were calculated by subtracting the post-virucidal efficacy test values for oraCMS1 and log_10_TCID_50_ values from the log_10_ titers obtained for the corresponding positive viral controls.

## Results

### Cytotoxicity, Sub-cytotoxicity, and Neutralization Tests

After neutralizing the CFS of oraCMS1, cytotoxicity was observed at the 10^−2^ dilution for Hep-2, MDCK, and CV-1 cells. No sub-cytotoxicity was observed in the test solution, thus confirming that the test solution was neutralized (Table 1).

**Table 1:** Results of sub-cytotoxicity and neutralization tests

**Dilution factor**	**RSV**		**H1N1**		**RVA**	
	**Sub-cytotoxicity**	**Neutralization**	**Sub-cytotoxicity**	**Neutralization**	**Sub-cytotoxicity**	**Neutralization**
Negative control	+	+	+	+	+	+
10^−1^	T	T	T	T	T	T
10^−2^	T	T	T	T	T	T
10^−3^	+	+	+	+	+	+
10^−4^	+	+	+	+	+	+
10^−5^	+	+	+	+	+	+
10^−6^	+	+	+	+	+	+
10^−7^	+	+	+	+	+	+
10^−8^	+	+	+	+	+	+
Judgment	No interference	Neutralized	No interference	Neutralized	No interference	Neutralized

*T* cytotoxicity, + viral infection, *RSV* respiratory syncytial virus, *RVA* rotavirus A, *H1N1* influenza A virus

### Virucidal Efficacy Test

**Fig. 1:**
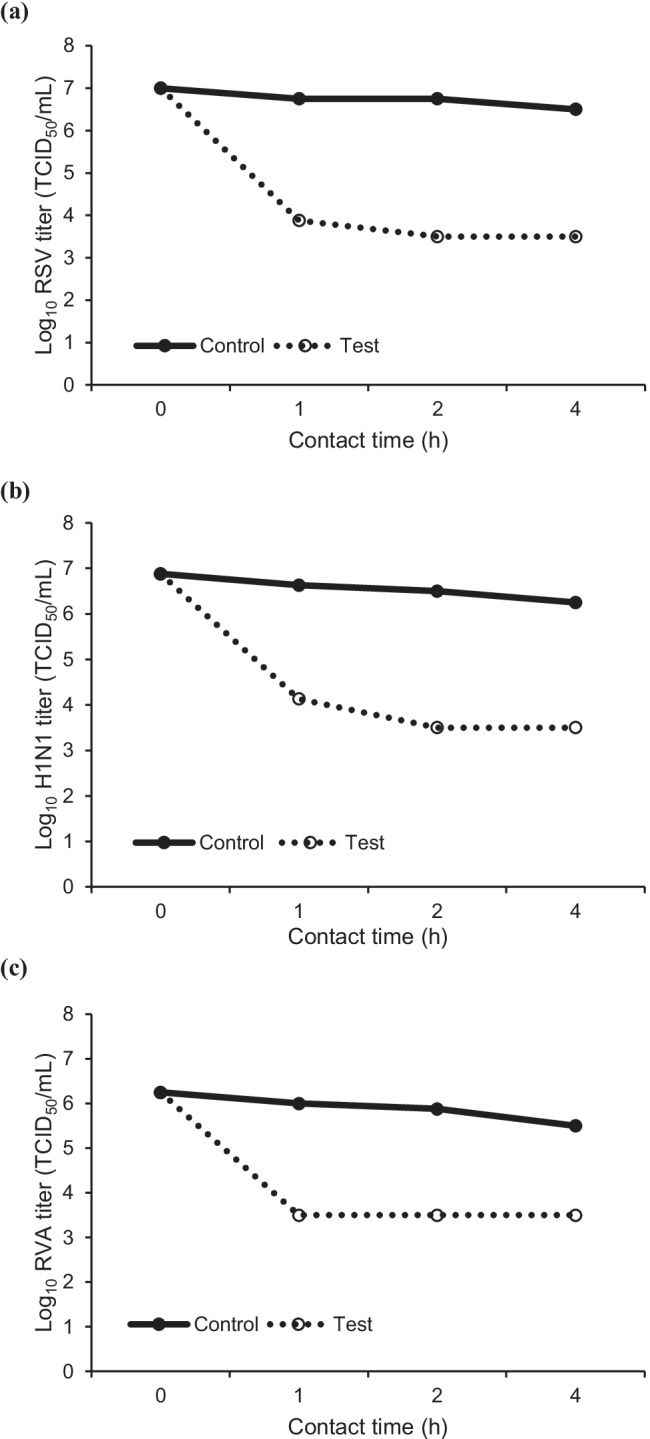
Efficacy of the cell-free supernatant of oraCMS1 in **a** respiratory syncytial virus (RSV), **b** influenza A virus (H1N1), and **c** rotavirus A (RVA) inactivation at various time points. Values represent representative mean values (*n* = 8 replicates) of the log_10_ titer of the positive control and test groups

The viral inactivation abilities of the CFS of oraCMS1 against RSV, H1N1, and RVA were determined. The samples from each incubation were titrated via the TCID_50_ endpoint assay using the appropriate host cell system for each virus. The tests were replicated eight times for each test solution, and for the viral recovery control, the mean values were reported. The efficacy of the CFS of oraCMS1 on RSV, H1N1, and RVA inactivation at various time points was presented (Fig. 1). The CFS of oraCMS1 at 2 h of contact time showed the best antiviral effect by inactivating RSV (99.9%) and H1N1 (99.9%) in vitro (Table 2).

**Table 2:** Inactivation of RSV, H1N1, and RVA by the cell-free supernatant of oraCMS1

	**Contact time (h)**	**RSV**	**H1N1**	**RVA**
Log reduction	1	2.87	2.50	≥2.50
	2	≥3.25	≥3.00	≥2.38
	4	≥3.00	≥2.75	≥2.00
Percentage (%) reduction	1	≥99.0	≥99.0	≥99.0
	2	≥99.9	≥99.9	≥99.0
	4	≥99.9	≥99.0	≥99.0

*RSV* respiratory syncytial virus, *RVA* rotavirus A, *H1N1* influenza A virus, *oraCMS1 Weissella cibaria* CMS1

## Discussion

The prevention of viral respiratory infections has become an important challenge in public health. RSV is a common RV that usually causes mild cold-like symptoms. Most people recover from infection within a week or two; however, RSV infections can be serious, particularly among infants and older adults. RSV is also recognized as the most common cause of acute lower respiratory infections, such as bronchiolitis and pneumonia in young children [7].

Probiotics may be an alternative and safe method to reduce the risk of infection in the absence of effective antiviral agents and vaccines against RSV [23–25]. To date, the most commonly studied anti-RV probiotics are *Lactobacillus* spp. One of the strains most frequently used in human application tests is the *Lactobacillus rhamnosus* strain, and most of the *Lactobacillus* strains can stimulate and strengthen the host immune system. In animal and human studies, *L. rhamnosus* has been reported to exert antiviral activity against RSV and H1N1. In RSV-infected mice, heat-killed *L. rhamnosus* CRL 1505 and CRL 1506 exerted an inhibitory effect on RSV by reducing the viral load in murine lungs due to IFN-α stimulation [28]. In addition, *L. rhamnosus* GG reduces the prevalence of upper and lower viral respiratory infections among children [29].

Recent studies have reported that *Lactobacillus plantarum* strains reduce the signs of influenza-like symptoms and can even increase the body weight and survival rate in mouse models. *L. plantarum* L-137, isolated from fermented foods, showed proinflammatory activity capable of reducing the titer of H1N1 in murine lungs [30]. Another strain, *L. plantarum* YU, showed anti-H1N1 activity by activating the Th1 immune response [31]. However, none of the *L. plantarum* strains has been evaluated in clinical trials, possibly owing to the secretion of undesirable acids or metabolites by this species. Most probiotics have been reported to be suppressed by reducing or releasing viral infectivity through immune regulatory mechanisms [28–31]. This is because RVs infect mucosal cells in the respiratory tract; thus, probiotics and their antimicrobial compounds cannot directly interact with the virus. However, these are interpreted as antiviral mechanisms of probiotics that act in the intestine.

oraCMS1 is a commercially available oral probiotic strain with proven safety [19] and inhibits the formation of a dental biofilm [18]. Our research group recently reported that the oraCMS1 exhibited antimicrobial activities against major pathogens of upper respiratory tract infections, including *Streptococcus pyogenes*, *Streptococcus pneumoniae*, and *Moraxella catarrhalis* [20]. Therefore, in the present study, the antiviral effects of oraCMS1 on RVs were also expected and investigated. According to the results presented in the present study, the CFS of oraCMS1 at 2 h of contact time showed the best antiviral effect by inactivating RSV (99.9%) and H1N1 (99.9%) in vitro.

RVA is the leading cause of severe enteritis in children younger than 5 years of age. Probiotics have been reported to prevent rotavirus infection, shorten the duration of RVA diarrhea, reduce the incidence of reinfection, and are supposedly related to the regulation of the immune response and viral excretion [32, 33]. In vitro and in vivo studies have shown that the mechanisms underlying the role of probiotics in RVA infection include the production of antimicrobial substances (lactic acid, hydrogen peroxide, short-chain fatty acids, and bacteriocin), mucin production by epithelial cells, and the innate immune response [34]. In our study, we investigated the antiviral effects of oraCMS1 on RVA. When contacted for 1, 2, or 4 h, oraCMS1 showed more than 99.0% antiviral activity against RVA.

As a decline in immunity increases susceptibility to periodontal disease and coronavirus disease (COVID-19), more caution is required. Patients with COVID-19 and periodontal diseases have an 8.81-fold higher mortality risk than other patients [35]. Periodontal disease worsens inflammation elsewhere in the body, which increases the risks of other diseases. In the presence of periodontal disease, oral bacteria can infect and worsen conditions of other organs. Thus, it is better to reduce the bacteria in the mouth by gargling with an oral antibacterial agent. Although oral antibacterial agents help to remove harmful bacteria, they also remove beneficial bacteria, thereby disrupting the balance in bacterial flora in the oral cavity. Therefore, in recent years, many safe methods for maintaining the balance of oral bacteria by ingesting oral probiotics have been introduced [18, 19]. In our previous study, we showed that *W. cibaria* has anti-inflammatory activity against periodontopathic pathogens; thus, it is expected to decrease susceptibility to COVID-19.

Several mechanisms underlying the antiviral activity of probiotics have been uncovered to date. First, the acidic pH produced by probiotics denatures the capsid protein of the virus to prevent cell adhesion. Second, the peptidoglycan structure in the cell wall of probiotics captures viral particles. Third, probiotic bacteria inhibit viral replication by preventing the virus from entering host cells via production and release of bacteriocin and hydrogen peroxide. Fourth, probiotics and viruses competitively attach to the host cell, and the former produce reactive oxygen species that kill the virus.

The probiotic strain used in our study is an oral probiotic that is expected to act locally in the oral cavity. In the present study, it was expected that the CFS of oraCMS1 would increase the antiviral efficacy, attributable to the inhibition of viral replication by substances contained in the fermented product. The oral cavity can act as the first path for infection by external pathogens such as viruses. As oraCMS1 resides in the mouth, it can prevent the invasion of these viruses.

The ingestion of the probiotic oraCMS1 is a promising strategy for the prevention of RV and RVA infections. The oraCMS1 used in this study is safe to ingest and could be effective in alleviating the symptoms associated with these viral infections. However, a limitation of our study was the inability to determine the detailed antiviral mechanisms by which oraCMS1 inhibits viral replication, which remains to be elucidated. Further studies will be needed to clarify which of the metabolites of oraCMS1 exhibited antiviral effect.

## Conclusions

Our study showed that oraCMS1 exerted antiviral effects against RSV, H1N1, and RVA in vitro. Thus, this probiotic can help prevent respiratory tract diseases and enteritis as oraCMS1 was shown to have a prophylactic potential against RV and RVA infections.

## Data Availability

The data presented in this study are available on request from the corresponding author.
